# Supplementary material for: Validation and Simultaneous Monitoring of 311 Pesticide Residues in Loamy Sand Agricultural Soils by LC-MS/MS and GC-MS/MS, Combined with QuEChERS-Based Extraction
Source: Molecules. 2023 May 23;28(11):4268. doi: 10.3390/molecules28114268 (PMC10254206; doi:10.3390/molecules28114268)
Supplement: Supplementary file 1 [file molecules-28-04268-s001.zip › Supplementary Table S1.docx]

**Table S1.** Data acquisition parameters and retention time for GC-MS/MS analysis. operated in multiple reaction monitoring (MRM) mode.

| **Analyte** | **Purity**  **(%)** | **RT**  **(min)** | **Quantification Ion**  **transitions** | **CE**  **(eV)** | **Qualification Ion**  **Transitions**  **I** | **CE**  **(eV)** | **Qualification Ion**  **Transitions**  **II** | **CE**  **(eV)** | **LOQ** | **Average Recoveries**  **(n=6)** | | | **RSD**  **(%)** | | |
| --- | --- | --- | --- | --- | --- | --- | --- | --- | --- | --- | --- | --- | --- | --- | --- |
|  |  |  |  |  |  |  |  |  |  | **10**  **ppb** | **20**  **ppb** | **100**  **ppb** | **10**  **ppb** | **20**  **ppb** | **100**  **ppb** |
| 2.4'-Methoxychlor | 99 | 17.62 | 227.1>121.1 | 16 | 121.1>78.0 | 22 | 121.1>91.0 | 12 | 0.02 | 94 | 95 | 93 | 6.8 | 5.2 | 5.6 |
| 4.4'-Dichlorobenzophenone | 99 | 14.33 | 139.0>111.0 | 14 | 139.0>75.0 | 26 | 249.9>139.0 | 16 | 0.01 | 110 | 88 | 86 | 7.1 | 11.9 | 11.8 |
| 4.4'-methoxychlor olefin | 94 | 17.28 | 238.1>223.1 | 12 | 308.0>238.1 | 16 | 238.1>195.1 | 20 | 0.01 | 94 | 88 | 93 | 5.5 | 2.8 | 6.8 |
| Acetochlor | 97 | 12.92 | 174.1>146.1 | 12 | 223.1>132.1 | 22 | 223.1>147.1 | 10 | 0.01 | 92 | 81 | 84 | 15.5 | 16.4 | 17.5 |
| Acrinathrin | 97 | 20.36 | 181.1>152.1 | 26 | 181.1>127.1 | 28 | - | - | 0.01 | 103 | 91 | 97 | 18.5 | 7.5 | 5.4 |
| Alachlor | 99 | 13.11 | 188.1>160.1 | 10 | 188.1>132.1 | 18 | 160.1>132.1 | 10 | 0.01 | 96 | 87 | 92 | 15.4 | 17.5 | 15.6 |
| alpha-BHC | 99 | 11.12 | 180.9>144.9 | 16 | 218.9>182.9 | 8 | 218.9>144.9 | 20 | 0.01 | 93 | 81 | 98 | 18.2 | 19.0 | 18.0 |
| alpha-Endosulfan | 99 | 15.55 | 194.9>160.0 | 8 | 194.9>125.0 | 24 | 194.9>123.0 | 22 | 0.1 | 97 | 74 | 91 | 17.2 | 17.3 | 12.1 |
| Ametryn | 98.3 | 13.33 | 227.1>185.1 | 6 | 227.1>58.0 | 14 | 227.1>170.1 | 14 | 0.02 | 105 | 93 | 92 | 16.5 | 17.9 | 17.0 |
| Azinphos-ethyl | 99 | 19.82 | 132.1>77.0 | 14 | 160.1>132.1 | 4 | 160.1>77.0 | 18 | 0.01 | 88 | 88 | 96 | 9.7 | 10.4 | 7.2 |
| Azinphos-methyl | 97 | 19.82 | 160.1>132.1 | 6 | 160.1>77.0 | 20 | 132.1>77.0 | 14 | 0.01 | 90 | 90 | 97 | 4.9 | 9.1 | 6.1 |
| Azoxystrobin | 99.5 | 23.25 | 344.1>329.1 | 16 | 388.1>345.1 | 18 | 388.1>300.1 | 20 | 0.01 | 90 | 83 | 96 | 9.1 | 6.0 | 7.2 |
| Benfluralin | 99 | 10.61 | 292.1>264.0 | 8 | 292.1>160.0 | 22 | 292.1>206.0 | 12 | 0.1 | 91 | 89 | 82 | 17.0 | 9.4 | 19.8 |
| beta-BHC | 99 | 11.69 | 180.9>144.9 | 16 | 218.9>182.9 | 8 | 218.9>144.9 | 20 | 0.02 | 104 | 75 | 88 | 12.0 | 16.3 | 16.9 |
| beta-Endosulfan | 99 | 16.75 | 194.9>123.0 | 22 | 194.9>160.0 | 8 | 194.9>125.0 | 24 | 0.01 | 110 | 106 | 90 | 13.4 | 14.3 | 8.5 |
| Bifenthrin | 99 | 18.35 | 181.0>165.0 | 23 | 181.1>166.1 | 12 | 181.1>179.1 | 12 | 0.01 | 96 | 93 | 95 | 4.2 | 3.5 | 6.5 |
| Boscalid | 99.5 | 21.25 | 140.1>112.1 | 12 | 140.1>76.0 | 24 | 342.1>140.1 | 14 | 0.01 | 85 | 82 | 90 | 5.0 | 2.0 | 6.4 |
| Bromfenvinfos-methyl | 97 | 14.85 | 294.9>109.0 | 16 | 296.9>109.0 | 16 | 109.0>79.0 | 3 | 0.01 | 102 | 91 | 98 | 10.9 | 9.2 | 13.2 |
| Bromfenvinphos | 98 | 15.59 | 266.9>159.0 | 14 | 268.9>161.0 | 16 | 322.9>266.9 | 12 | 0.01 | 94 | 85 | 96 | 10.1 | 9.3 | 10.4 |
| Bromophos methyl | 99 | 14.39 | 331.0>316.0 | 11 | 331.0>286.0 | 24 | 329.0>314.0 | 11 | 0.01 | 83 | 99 | 91 | 14.8 | 10.3 | 13.2 |
| Bromophos-ethyl | 98 | 15.19 | 358.9>302.9 | 16 | 357.0>301.0 | 13 | 331.0>301.0 | 6 | 0.01 | 96 | 84 | 94 | 6.2 | 11.6 | 16.0 |
| Bromopropylate | 99 | 18.46 | 183.0>155.0 | 20 | 340.9>182.9 | 18 | 340.9>184.9 | 20 | 0.02 | 95 | 94 | 92 | 4.8 | 4.0 | 5.7 |
| Bupirimate | 99 | 16.06 | 273.1>108.1 | 16 | 273.1>193.1 | 8 | 316.1>208.1 | 10 | 0.01 | 97 | 92 | 96 | 7.7 | 3.7 | 9.7 |
| Carbophenothion | 99 | 17.31 | 157.0>45.0 | 18 | 125.0>97.0 | 4 | 341.9>157.0 | 14 | 0.01 | 95 | 90 | 94 | 3.3 | 3.6 | 7.8 |
| Carboxin | 99.5 | 16.21 | 143.0>87.0 | 8 | 235.1>143.0 | 12 | 235.1>87.0 | 24 | 0.01 | 81 | 85 | 91 | 15.0 | 5.0 | 8.2 |
| Carfentrazone-ethyl | 97 | 17.17 | 340.1>312.1 | 14 | 330.0>310.0 | 6 | 312.1>151.1 | 24 | 0.01 | 95 | 90 | 96 | 8.8 | 3.5 | 7.3 |
| Chlorbenside | 99 | 15.33 | 125.0>89.0 | 16 | 125.0>99.0 | 18 | 127.0>89.0 | 18 | 0.01 | 80 | 87 | 88 | 16.8 | 14.2 | 12.1 |
| Chlorfenapyr | 99 | 16.23 | 247.1>227.0 | 16 | 139.0>102.0 | 12 | 247.1>200.0 | 24 | 0.01 | 100 | 109 | 93 | 14.7 | 11.1 | 12.2 |
| Chlorfenson | 99 | 15.81 | 175.0>111.0 | 12 | 175.0>75.0 | 28 | 301.9>175.0 | 8 | 0.01 | 90 | 89 | 92 | 7.7 | 10.0 | 7.1 |
| Chlorfenvinphos | 97 | 15.59 | 267.0>159 | 13 | 323.0>267.0 | 8 | 295.0>267.0 | 3 | 0.01 | 96 | 89 | 97 | 10.4 | 5.4 | 12.1 |
| Chlorobenzilate | 99 | 16.59 | 139.0>111.0 | 16 | 251.0>139.0 | 14 | 139.0>75.0 | 26 | 0.01 | 95 | 88 | 93 | 4.4 | 5.2 | 7.0 |
| Chlorpyrifos | 99 | 13.92 | 196.9>168.9 | 14 | 313.9>257.9 | 14 | 313.9>285.9 | 8 | 0.01 | 106 | 86 | 84 | 12.5 | 10.4 | 18.1 |
| Chlorpyrifos-methyl | 98 | 12.98 | 285.9>93.0 | 22 | 287.9>93.0 | 22 | 285.9>270.9 | 14 | 0.01 | 97 | 83 | 90 | 16.0 | 11.2 | 18.9 |
| Chlorthal-dimethyl | 99 | 14.01 | 298.9>220.9 | 24 | 300.9>222.9 | 26 | 300.9>272.9 | 14 | 0.01 | 92 | 80 | 92 | 13.3 | 14.5 | 14.3 |
| Chlorthiophos | 98 | 16.80 | 324.9>268.9 | 14 | 268.9>205.0 | 18 | 297.0>269.0 | 6 | 0.01 | 95 | 88 | 96 | 5.8 | 6.1 | 7.1 |
| Chlozolinate | 99 | 14.74 | 186.0>145.0 | 14 | 330.9>258.9 | 6 | 258.9>188.0 | 14 | 0.01 | 83 | 70 | 92 | 11.7 | 15.4 | 8.6 |
| cis-Chlordane | 99 | 15.52 | 374.8>265.9 | 26 | 372.8>263.9 | 28 | 372.8>265.9 | 22 | 0.01 | 91 | 88 | 89 | 16.6 | 14.4 | 14.5 |
| cis-Nonachlor | 99 | 16.77 | 406.8>299.9 | 24 | 406.8>109.0 | 22 | 406.8>334.9 | 16 | 0.1 | 98 | 95 | 87 | 12.0 | 5.6 | 5.8 |
| cis-Permethrine | 99 | 20.23 | 183.1>153.1 | 14 | 183.1>168.1 | 14 | 183.1>165.1 | 10 | 0.01 | 94 | 88 | 96 | 5.5 | 2.2 | 5.7 |
| Clomazone | 99 | 11.69 | 204.1>107.0 | 20 | 204.1>78.0 | 26 | 204.1>68.0 | 24 | 0.01 | 82 | 78 | 87 | 11.9 | 19.2 | 18.9 |
| Coumaphos | 98 | 20.39 | 362.0>109.0 | 16 | 210.0>182.0 | 20 | 362.0>226.0 | 14 | 0.01 | 93 | 85 | 99 | 7.2 | 11.6 | 8.4 |
| Cycloate | 95 | 10.36 | 154.2>83.1 | 8 | 154.2>55.0 | 18 | 154.2>72.0 | 6 | 0.01 | 105 | 104 | 101 | 18.0 | 11.6 | 16.3 |
| Cyfluthrin | 99 | 20.87 | 163.1>127.1 | 6 | 163.1>91.0 | 14 | 226.1>206.1 | 14 | 0.01 | 77 | 76 | 88 | 12.4 | 14.0 | 8.7 |
| Cypermethrin | 99 | 21.21 | 163.1>127.1 | 6 | 163.1>91.0 | 14 | 127.0>91.0 | 6 | 0.01 | 89 | 78 | 90 | 5.3 | 18.6 | 7.9 |
| Cyprodinil | 99 | 14.66 | 224.1>208.1 | 16 | 224.1>197.1 | 22 | 224.1>131.1 | 14 | 0.01 | 85 | 76 | 96 | 17.2 | 14.2 | 16.5 |
| delta-BHC | 98 | 12.49 | 180.9>144.9 | 16 | 218.9>182.9 | 8 | 218.9>144.9 | 20 | 0.01 | 99 | 101 | 79 | 18.2 | 15.8 | 17.6 |
| Deltamethrin | 99 | 22.98 | 180.9>151.9 | 22 | 252.9>93.0 | 20 | 252.9>171.9 | 8 | 0.01 | 75 | 75 | 91 | 14.4 | 19.1 | 14.1 |
| Di-allate | 95 | 10.95 | 234.1>150.0 | 20 | 234.1>192.1 | 14 | 128.0>86.0 | 4 | 0.01 | 103 | 72 | 82 | 14.9 | 18.7 | 13.9 |
| Diazinon | 98 | 11.97 | 137.0>84.0 | 12 | 199.0>93.0 | 16 | 179.1>137.1 | 18 | 0.01 | 118 | 99 | 89 | 7.6 | 15.3 | 18.6 |
| Dichlobenil | 98 | 7.2 | 170.9>100.0 | 24 | 170.9>136 | 14 | 170.9>110.0 | 14 | 0.01 | 113 | 73 | 89 | 12.4 | 16.1 | 12.0 |
| Dichlofluanid | 99 | 13.78 | 123.0>77.0 | 16 | 223.9>123.1 | 8 | 167.1>124.1 | 10 | 0.01 | 79 | 70 | 77 | 11.4 | 9.1 | 16.8 |
| Dieldrin | 98 | 16.08 | 262.9>193.0 | 34 | 276.9>241.0 | 8 | 262.9>228.0 | 24 | 0.01 | 102 | 88 | 88 | 15.9 | 11.6 | 13.7 |
| Difenoconazole | 99.5 | 22.64 | 323.0>265.0 | 14 | 265.0>202.0 | 20 | 265.0>139.0 | 30 | 0.01 | 91 | 84 | 92 | 6.8 | 3.3 | 6.2 |
| Diflufenican | 99.8 | 17.78 | 394.1>266.0 | 14 | 266.0>246.0 | 14 | 266.0>218.0 | 24 | 0.01 | 96 | 93 | 97 | 7.2 | 5.8 | 6.4 |
| Dimethachlor | 99 | 12.86 | 197.1>148.1 | 10 | 197.1>120.1 | 22 | 199.1>148.1 | 10 | 0.01 | 102 | 83 | 95 | 11.3 | 13.2 | 17.0 |
| Dimethenamid (Dimethenamid-P) | 99 | 12.86 | 230.0>154.1 | 10 | 203.0>126.1 | 22 | 203.0>154.1 | 12 | 0.01 | 93 | 94 | 88 | 5.5 | 18.5 | 18.8 |
| Dimethomorph | 99.2 | 23.4 | 301.1>165.1 | 14 | 387.1>301.1 | 12 | 301.1>139.0 | 14 | 0.01 | 77 | 82 | 95 | 4.0 | 2.0 | 6.7 |
| Diphenamid | 99 | 14.41 | 167.1>152.1 | 20 | 239.1>167.1 | 8 | 239.1>72.0 | 16 | 0.01 | 96 | 89 | 94 | 11.8 | 12.5 | 9.7 |
| Disulfoton | 97 | 12.24 | 88.0>60.0 | 4 | 153.0>97.0 | 10 | 153.0>125.0 | 6 | 0.01 | 95 | 86 | 92 | 14.1 | 12.5 | 16.0 |
| Edifenphos | 98 | 17.39 | 173.0>109.0 | 10 | 109.0>65.0 | 11 | 310.0>109.0 | 26 | 0.01 | 97 | 90 | 101 | 9.0 | 7.6 | 8.1 |
| Endosulfan sulfate | 99 | 17.48 | 271.8>236.9 | 18 | 386.8>252.9 | 16 | 386.8>288.8 | 10 | 0.01 | 93 | 91 | 90 | 6.7 | 8.2 | 7.8 |
| Endrin | 99 | 16.51 | 262.9>191.0 | 30 | 262.9>193.0 | 28 | 244.9>173.0 | 32 | 0.01 | 101 | 91 | 90 | 12.2 | 14.6 | 9.7 |
| Endrin ketone | 99 | 18.42 | 316.9>244.9 | 20 | 314.9>242.9 | 18 | 316.9>101.0 | 16 | 0.01 | 84 | 95 | 91 | 18.2 | 11.5 | 5.9 |
| EPN | 98 | 18.46 | 169.1>140.9 | 8 | 156.9>77.0 | 24 | 169.1>77.0 | 22 | 0.01 | 95 | 95 | 94 | 6.4 | 6.3 | 5.5 |
| Epoxiconazole | 98.7 | 18.06 | 192.0>138.0 | 14 | 192.0>111.0 | 26 | 192.0>165.0 | 8 | 0.01 | 97 | 94 | 94 | 2.0 | 1.7 | 6.2 |
| Ethion | 99 | 16.75 | 153.0>97.0 | 14 | 230.9>129.0 | 24 | 153.0>125.0 | 6 | 0.01 | 92 | 92 | 98 | 11.2 | 6.2 | 8.4 |
| Ethoprophos | 99.1 | 10.34 | 200.0>158.0 | 6 | 158.0>97.0 | 18 | 158.0>114.0 | 8 | 0.01 | 95 | 89 | 95 | 15.7 | 16.7 | 18.0 |
| Etofenprox | 97 | 21.45 | 163.1>135.1 | 10 | 163.1>107.1 | 18 | 135.1>107.1 | 10 | 0.01 | 87 | 84 | 93 | 3.2 | 2.6 | 5.9 |
| Famoxadone | 99.4 | 23.55 | 224.1>196.1 | 10 | 330.1>224.1 | 10 | 330.1>196.1 | 22 | 0.01 | 81 | 83 | 91 | 12.1 | 11.7 | 5.5 |
| Fenamiphos | 99 | 15.64 | 303.1>195.1 | 8 | 288.1>260.1 | 6 | 303.1>154.1 | 18 | 0.01 | 89 | 85 | 98 | 13.9 | 15.2 | 12.5 |
| Fenarimol | 99 | 19.67 | 251.0>139.0 | 14 | 330.0>139.0 | 8 | 251.0>111.0 | 26 | 0.01 | 92 | 91 | 95 | 7.6 | 3.4 | 8.2 |
| Fenchlorphos | 98 | 13.33 | 284.9>269.9 | 16 | 286.9>271.9 | 18 | 284.9>239.9 | 26 | 0.01 | 103 | 92 | 90 | 13.5 | 11.0 | 19.3 |
| Fenoxaprop-ethyl (Fenoxaprop-P-ethyl) | 98 | 19.99 | 361.1>288.1 | 12 | 288.1>119.1 | 12 | 288.1>91.0 | 20 | 0.1 | 89 | 92 | 91 | 8.9 | 5.6 | 5.6 |
| Fenoxycarb | 99.5 | 18.55 | 255.1>186.1 | 10 | 186.1>109.1 | 14 | 186.1>129.1 | 14 | 0.01 | 93 | 74 | 92 | 4.6 | 18.2 | 7.8 |
| Fenpropathrin | 99 | 18.58 | 181.1>152.1 | 22 | 265.1>210.1 | 12 | 181.1>127.1 | 28 | 0.01 | 101 | 96 | 94 | 1.6 | 3.4 | 5.1 |
| Fenson | 99 | 14.44 | 141.0>77.0 | 16 | 267.9>141.0 | 6 | 267.9>77.0 | 20 | 0.01 | 90 | 83 | 94 | 4.7 | 17.9 | 12.8 |
| Fenthion | 99 | 14.06 | 278.0>109.0 | 20 | 278.0>125.0 | 20 | 278.0>169.0 | 14 | 0.02 | 96 | 87 | 94 | 8.1 | 13.8 | 15.8 |
| Fenvalerate | 99 | 22.06 | 167.0>125.0 | 6 | 169.0>127.0 | 8 | 225.1>119.1 | 20 | 0.01 | 79 | 77 | 92 | 12.5 | 16.7 | 8.0 |
| Fipronil | 97 | 14.62 | 366.9>212.9 | 30 | 368.9>214.9 | 30 | 366.9>254.9 | 22 | 0.01 | 93 | 86 | 95 | 12.3 | 7.1 | 7.5 |
| Fluazifop-P-butyl | 98 | 16.39 | 282.1>238.1 | 18 | 282.1>91.0 | 18 | 383.1>282.1 | 14 | 0.01 | 103 | 86 | 98 | 16.5 | 7.4 | 7.6 |
| Fluchloralin | 99 | 11.97 | 306.0>264.0 | 8 | 326.0>63.0 | 16 | 328.0>65.0 | 16 | 0.01 | 102 | 83 | 84 | 14.8 | 9.3 | 14.9 |
| Flucythrinate | 94 | 21.26 | 199.1>157.1 | 10 | 157.1>107.1 | 12 | 199.1>107.1 | 22 | 0.02 | 85 | 78 | 92 | 6.5 | 13.0 | 7.6 |
| Fludioxonil | 99 | 15.85 | 248.0>127.0 | 26 | 248.0>154.0 | 20 | 182.0>127.0 | 16 | 0.01 | 99 | 92 | 97 | 8.7 | 16.4 | 8.7 |
| Flufenacet | 97.8 | 14.08 | 151.0>136.0 | 12 | 151.0>95.0 | 26 | 210.9>123.0 | 10 | 0.01 | 87 | 88 | 95 | 9.9 | 13.3 | 17.7 |
| Fluquinconazole | 99 | 20.40 | 340.0>298.0 | 20 | 340.0>313.0 | 14 | 342.0>300.0 | 22 | 0.01 | 92 | 87 | 95 | 7.5 | 6.2 | 4.5 |
| Fluridone | 99 | 21.73 | 328.1>259.0 | 24 | 328.1>313.0 | 22 | 328.1>127.0 | 24 | 0.01 | 88 | 84 | 98 | 5.6 | 7.8 | 7.7 |
| Flusilazole | 98 | 16.1 | 233.1>165.1 | 14 | 206.1>151.1 | 16 | 233.1>152.1 | 14 | 0.01 | 101 | 82 | 98 | 6.3 | 14.0 | 10.9 |
| Flutolanil | 99 | 15.7 | 173.0>145.0 | 14 | 173.0>95.0 | 26 | 281.1>173.0 | 12 | 0.01 | 98 | 95 | 96 | 6.7 | 4.5 | 8.6 |
| Flutriafol | 98 | 15.62 | 219.1>123.1 | 14 | 219.1>95.0 | 28 | 164.1>95.0 | 28 | 0.01 | 90 | 90 | 96 | 6.0 | 8.2 | 13.0 |
| Fonofos | 97 | 11.91 | 137.1>109.1 | 8 | 246.0>137.1 | 6 | 246.0>109.1 | 18 | 0.01 | 100 | 82 | 95 | 14.6 | 11.9 | 17.7 |
| gamma-BHC (Lindane) | 99 | 11.83 | 180.9>144.9 | 16 | 218.9>182.9 | 8 | 218.9>144.9 | 20 | 0.01 | 97 | 93 | 83 | 16.3 | 16.9 | 18.7 |
| Hexachlorobenzene | 99 | 11.15 | 283.8>248.8 | 24 | 283.8>213.8 | 28 | 285.8>250.8 | 22 | 0.01 | 87 | 74 | 87 | 17.3 | 14.8 | 13.9 |
| Hexazinone | 99 | 17.65 | 171.1>71.0 | 16 | 171.1>85.0 | 16 | 128.1>83.0 | 10 | 0.01 | 97 | 101 | 100 | 4.4 | 5.2 | 5.8 |
| Indoxacarb | 99.9 | 22.74 | 218.0>203.0 | 10 | 264.0>176.0 | 14 | 218.0>134.0 | 22 | 0.01 | 81 | 77 | 97 | 14.5 | 11.9 | 17.7 |
| Iodofenphos | 98 | 15.74 | 376.9>361.8 | 22 | 376.9>331.8 | 32 | 378.9>363.8 | 24 | 0.01 | 89 | 91 | 94 | 10.9 | 4.2 | 10.0 |
| Isazofos | 99 | 12.25 | 161.0>119.0 | 8 | 172.0>130.0 | 8 | 161.0>146.0 | 6 | 0.01 | 89 | 98 | 87 | 15.3 | 15.3 | 16.1 |
| Isodrin | 99 | 14.62 | 192.9>157.0 | 20 | 192.9>123.0 | 26 | 262.9>192.9 | 28 | 0.01 | 89 | 82 | 93 | 12.9 | 14.6 | 13.4 |
| Isopropalin | 98 | 14.44 | 280.1>238.1 | 8 | 280.1>133.1 | 18 | 280.1>165.1 | 16 | 0.01 | 103 | 90 | 86 | 15.9 | 12.2 | 14.4 |
| Kresoxim-methyl | 99.9 | 16.09 | 206.1>131.1 | 14 | 206.1>116.1 | 6 | 131.1>89.0 | 28 | 0.01 | 93 | 88 | 94 | 9.6 | 0.8 | 6.8 |
| lambda-Cyhalothrin | 99 | 19.36 | 181.1>152.1 | 24 | 208.0>181.0 | 6 | 197.0>141.0 | 8 | 0.01 | 86 | 82 | 91 | 10.6 | 12.1 | 8.7 |
| Lenacil | 99 | 17.51 | 153.1>136.1 | 14 | 153.1>82.1 | 16 | 153.1>110.1 | 16 | 0.02 | 94 | 103 | 105 | 9.8 | 10.2 | 8.3 |
| Leptophos | 98 | 19.1 | 171.0>77.0 | 16 | 376.9>361.9 | 24 | 171.0>124.0 | 6 | 0.01 | 90 | 90 | 94 | 6.9 | 3.2 | 5.9 |
| Malathion | 98 | 13.79 | 173.1>127.0 | 6 | 173.1>99.0 | 14 | 158.1>125.0 | 10 | 0.02 | 98 | 85 | 93 | 8.0 | 15.1 | 15.6 |
| Mefenpyr-diethyl | 99.7 | 18.04 | 253.0>189.0 | 28 | 299.0>253.0 | 10 | 253.0>163.0 | 24 | 0.02 | 95 | 94 | 94 | 9.6 | 4.2 | 6.9 |
| Metalaxyl (Mefenoxam) | 99 | 13.27 | 206.1>132.1 | 20 | 249.2>190.1 | 8 | 160.0>145.0 | 8 | 0.1 | 92 | 87 | 91 | 8.9 | 15.6 | 12.7 |
| Metazachlor | 99 | 14.67 | 209.1>132.1 | 18 | 133.1>117.1 | 24 | 211.1>132.1 | 20 | 0.01 | 89 | 85 | 93 | 11.9 | 10.6 | 14.7 |
| Methoxychlor | 98 | 18.57 | 227.1>169.1 | 24 | 227.1>212.1 | 14 | 227.1>141.1 | 28 | 0.01 | 94 | 89 | 94 | 4.5 | 3.7 | 6.0 |
| Metolachlor (S-Metolachlor) | 99 | 13.87 | 162.1>133.1 | 16 | 238.1>162.1 | 12 | 238.1>133.1 | 26 | 0.01 | 95 | 82 | 95 | 8.3 | 16.7 | 14.0 |
| Metribuzin | 93.80 | 13.05 | 198.1>82.0 | 14 | 198.1>110.1 | 10 | 144.1>128.1 | 8 | 0.01 | 88 | 91 | 86 | 11.7 | 16.8 | 16.2 |
| MGK 264 | 99 | 14.41 | 164.1>93.0 | 10 | 111.1>82.0 | 8 | 164.1>80.0 | 24 | 0.1 | 97 | 88 | 92 | 3.4 | 8.9 | 12.1 |
| Mirex | 99 | 19.56 | 271.8>236.8 | 18 | 273.8>238.8 | 18 | 271.8>234.8 | 18 | 0.01 | 88 | 91 | 88 | 2.5 | 3.2 | 5.0 |
| Myclobutanil | 99 | 16.06 | 179.1>125.0 | 14 | 179.1>152.0 | 8 | 150.0>123.0 | 18 | 0.01 | 97 | 89 | 100 | 5.0 | 5.2 | 7.7 |
| Napropamide | 98 | 15.68 | 128.1>72.0 | 6 | 100.0>72.0 | 8 | 128.1>100.0 | 8 | 0.01 | 100 | 86 | 96 | 13.1 | 7.5 | 13.8 |
| Nitralin | 99 | 17.9 | 316.1>274.0 | 8 | 274.0>169.0 | 12 | 274.0>216.0 | 8 | 0.01 | 97 | 93 | 93 | 8.8 | 6.0 | 5.9 |
| Nitrofen | 98 | 16.49 | 202.0>139.0 | 24 | 282.9>253.0 | 12 | 282.9>162.0 | 24 | 0.01 | 94 | 96 | 91 | 6.8 | 6.3 | 7.2 |
| Norflurazon | 99 | 17.38 | 303.0>145.0 | 22 | 145.0>95.0 | 18 | 145.0>75.0 | 28 | 0.01 | 89 | 95 | 98 | 10.5 | 14.7 | 9.6 |
| o.p'-DDD | 97 | 16.09 | 235.0>165.0 | 24 | 237.0>165.0 | 28 | 235.0>199.0 | 16 | 0.01 | 98 | 89 | 91 | 7.8 | 6.7 | 7.8 |
| o.p'-DDE | 99 | 15.31 | 246.0>176.0 | 30 | 248.0>176.0 | 28 | 246.0>211.0 | 22 | 0.1 | 89 | 83 | 90 | 9.7 | 12.8 | 9.0 |
| o.p'-DDT | 98 | 16.81 | 235.0>165.0 | 21 | 237.0>165.0 | 22 | 235.0>199.0 | 16 | 0.01 | 96 | 88 | 91 | 3.7 | 3.2 | 8.4 |
| Oxadiazon | 99 | 15.91 | 258.0>175.0 | 8 | 302.0>175.0 | 14 | 258.0>112.0 | 28 | 0.01 | 93 | 88 | 95 | 6.4 | 5.5 | 8.0 |
| Oxyfluorfen | 97 | 16.04 | 252.0>196.0 | 22 | 300.0>223.0 | 15 | 361.0>317.0 | 6 | 0.01 | 98 | 74 | 89 | 16.1 | 17.7 | 8.9 |
| p.p'-DDD | 99 | 16.81 | 235.0>165.0 | 24 | 237.0>165.0 | 22 | 235.0>199.0 | 12 | 0.01 | 94 | 87 | 92 | 6.6 | 4.6 | 6.7 |
| p.p'-DDE | 99 | 15.96 | 246.0>176.0 | 30 | 317.9>248.0 | 24 | 246.0>211.0 | 22 | 0.02 | 87 | 85 | 90 | 7.8 | 5.7 | 8.6 |
| p.p'-DDT | 99 | 17.51 | 235.0>165.0 | 24 | 237.0>165.0 | 28 | 235.0>199.0 | 16 | 0.01 | 95 | 91 | 94 | 4.0 | 4.3 | 7.5 |
| Paclobutrazol | 99 | 15.39 | 236.1>125.0 | 14 | 236.1>167.0 | 10 | 236.1>132.0 | 16 | 0.01 | 109 | 90 | 95 | 8.8 | 3.8 | 12.7 |
| Parathion-methyl | 98 | 13.15 | 125.0>47.0 | 12 | 125.0>79.0 | 8 | 263.0>109.0 | 14 | 0.01 | 106 | 87 | 106 | 10.4 | 15.5 | 16.9 |
| Penconazole | 99 | 14.74 | 248.1>157.1 | 26 | 159.1>123.1 | 22 | 248.1>192.1 | 14 | 0.01 | 101 | 87 | 92 | 11.1 | 9.7 | 13.2 |
| Pendimethalin | 99 | 14.58 | 252.1>162.1 | 10 | 252.1>191.1 | 8 | 252.1>208.1 | 6 | 0.01 | 105 | 90 | 85 | 16.9 | 14.8 | 12.1 |
| Pentachloroaniline | 98 | 12.75 | 262.9>191.9 | 22 | 264.9>193.9 | 18 | 264.9>191.9 | 18 | 0.01 | 103 | 119 | 93 | 14.8 | 8.7 | 15.0 |
| Pentachloroanisole | 99 | 11.26 | 264.8>236.8 | 16 | 279.9>236.8 | 26 | 279.9>264.8 | 12 | 0.01 | 77 | 87 | 90 | 17.8 | 18.8 | 17.1 |
| Pentachlorobenzene | 99 | 9.08 | 249.9>214.9 | 18 | 249.9>178.9 | 28 | 249.9>176.9 | 26 | 0.01 | 72 | 74 | 81 | 6.3 | 9.7 | 16.6 |
| Pentachlorothioanisole | 98 | 13.73 | 295.8>262.9 | 14 | 295.8>245.8 | 30 | 297.8>264.9 | 16 | 0.01 | 76 | 108 | 95 | 16.6 | 16.3 | 15.6 |
| Perthane | 99 | 16.45 | 223.0>167.0 | 12 | 223.0>179.0 | 12 | 223.0>193.0 | 28 | 0.01 | 96 | 88 | 94 | 3.8 | 2.6 | 8.2 |
| Phenothrin | 93 | 18.96 | 123.1>81.0 | 8 | 183.1>153.1 | 14 | 183.1>168.1 | 14 | 0.01 | 95 | 87 | 97 | 6.5 | 7.9 | 6.1 |
| Phorate | 97 | 10.98 | 121.0>65.0 | 8 | 121.0>93.0 | 4 | 260.0>75.0 | 8 | 0.01 | 93 | 87 | 88 | 14.5 | 12.1 | 16.3 |
| Phosalone | 99 | 19.1 | 182.0>111.0 | 14 | 182.0>138.0 | 8 | 182.0>102.0 | 14 | 0.01 | 94 | 93 | 95 | 8.2 | 5.7 | 5.9 |
| Phosmet | 98 | 18.47 | 160.0>77.0 | 24 | 160.0>133.0 | 14 | 160.0>105.0 | 18 | 0.01 | 89 | 90 | 94 | 13.2 | 11.6 | 8.3 |
| Piperonyl butoxide | 94 | 17.88 | 176.1>131.1 | 12 | 176.1>117.1 | 20 | 176.1>103.1 | 24 | 0.01 | 93 | 95 | 97 | 4.2 | 1.8 | 5.9 |
| Pirimiphos ethyl | 99 | 14.33 | 304.1>168.1 | 12 | 318.1>166.1 | 12 | 318.1>182.1 | 12 | 0.01 | 98 | 86 | 94 | 13.2 | 7.1 | 16.7 |
| Pirimiphos-methyl | 99 | 13.54 | 290.1>125.0 | 22 | 290.1>233.1 | 12 | 305.1>180.1 | 8 | 0.01 | 99 | 80 | 96 | 14.8 | 11.3 | 14.5 |
| Pretilachlor | 96 | 15.77 | 262.1>202.1 | 10 | 238.1>162.1 | 10 | 238.1>146.1 | 10 | 0.01 | 91 | 87 | 94 | 11.8 | 7.4 | 10.5 |
| Prochloraz | 99 | 20.44 | 180.1>138.1 | 12 | 180.1>69.0 | 20 | 180.1>95.0 | 20 | 0.01 | 82 | 91 | 99 | 8.5 | 8.2 | 6.6 |
| Procymidone | 99 | 15.01 | 283.0>96.0 | 10 | 285.0>96.0 | 10 | 283.0>68.0 | 24 | 0.1 | 88 | 93 | 98 | 14.4 | 4.1 | 11.5 |
| Prodiamine | 99 | 13.56 | 321.1>279.1 | 6 | 321.1>203.1 | 10 | 321.1>205.1 | 14 | 0.01 | 92 | 88 | 91 | 8.1 | 13.3 | 15.9 |
| Profenofos | 98 | 15.89 | 336.9>266.9 | 14 | 338.9>268.9 | 18 | 339.0>188.0 | 28 | 0.02 | 113 | 79 | 102 | 9.6 | 14.4 | 6.7 |
| Prometryn | 99.8 | 13.37 | 226.1>184.1 | 10 | 241.2>184.1 | 12 | 241.2>58.0 | 14 | 0.01 | 94 | 85 | 91 | 15.5 | 11.3 | 16.0 |
| Propachlor | 98 | 10.04 | 120.0>77.0 | 20 | 176.1>57.0 | 8 | 120.0>92.0 | 8 | 0.01 | 114 | 93 | 92 | 8.0 | 16.3 | 18.8 |
| Propargite | 99 | 17.79 | 135.1>107.1 | 16 | 135.1>77.0 | 24 | 135.1>95.0 | 14 | 0.01 | 97 | 92 | 101 | 7.7 | 2.6 | 5.0 |
| Propisochlor | 96 | 13.18 | 162.1>120.1 | 14 | 162.1>147.1 | 14 | 162.1>144.1 | 12 | 0.01 | 84 | 86 | 91 | 12.3 | 7.1 | 19.6 |
| Propyzamide | 97 | 11.95 | 172.9>144.9 | 16 | 172.9>109.0 | 26 | 172.9>74.0 | 28 | 0.01 | 96 | 81 | 90 | 11.5 | 16.5 | 17.0 |
| Prothiofos | 97 | 15.77 | 266.9>238.9 | 10 | 309.0>238.9 | 14 | 266.9>220.9 | 20 | 0.02 | 94 | 85 | 92 | 15.0 | 9.7 | 9.4 |
| Pyraclofos | 99 | 19.94 | 194.0>138.0 | 22 | 360.1>194.0 | 14 | 360.1>139.0 | 14 | 0.01 | 92 | 84 | 101 | 7.8 | 12.4 | 6.4 |
| Pyrazophos | 99 | 19.58 | 221.1>193.1 | 12 | 221.1>149.1 | 14 | 221.1>177.1 | 16 | 0.01 | 93 | 89 | 96 | 5.6 | 4.0 | 6.3 |
| Pyridaben | 99 | 20.42 | 147.1>117.1 | 22 | 147.1>132.1 | 14 | 147.1>119.1 | 10 | 0.01 | 91 | 89 | 96 | 5.6 | 5.0 | 5.9 |
| Pyridaphenthion | 99 | 18.26 | 340.0>199.1 | 8 | 199.1>92.0 | 16 | 199.1>77.0 | 24 | 0.01 | 84 | 101 | 97 | 11.3 | 9.7 | 7.7 |
| Pyriproxyfen | 99 | 19.26 | 136.1>78.0 | 20 | 136.1>96.0 | 14 | 226.1>186.1 | 14 | 0.01 | 89 | 88 | 95 | 6.5 | 2.5 | 6.3 |
| Quinalphos | 99 | 14.96 | 146.1>118.0 | 10 | 146.1>91.0 | 24 | 157.1>129.0 | 14 | 0.01 | 94 | 82 | 95 | 16.3 | 9.8 | 12.1 |
| Quizalofop-ethyl (Quizalofop-P-ethyl) | 99 | 21.32 | 372.1>299.1 | 14 | 299.1>255.1 | 18 | 299.1>91.1 | 22 | 0.01 | 88 | 83 | 89 | 7.8 | 4.7 | 6.8 |
| Resmethrin (Bioresmethrin) | 99 | 17.94 | 143.1>128.1 | 10 | 171.1>143.1 | 6 | 123.0>81.0 | 6 | 0.01 | 92 | 82 | 88 | 7.2 | 8.7 | 6.5 |
| Sulfotep | 97 | 10.71 | 322.0>202.0 | 10 | 202.0>146.0 | 8 | 322.0>174.0 | 18 | 0.01 | 94 | 84 | 89 | 13.7 | 14.3 | 18.5 |
| Sulprofos | 98 | 17.09 | 156.0>141.0 | 18 | 156.0>108.0 | 28 | 322.0>156.0 | 8 | 0.01 | 101 | 90 | 94 | 5.7 | 6.8 | 8.3 |
| tau-Fluvalinate | 97 | 22.13 | 250.1>55.0 | 18 | 250.1>200.1 | 16 | 252.0>55.0 | 15 | 0.01 | 79 | 70 | 90 | 17.8 | 8.1 | 15.8 |
| Tebuconazole | 99 | 17.79 | 250.1>125.1 | 22 | 125.1>89.0 | 18 | 250.1>153.1 | 12 | 0.01 | 95 | 97 | 100 | 9.0 | 3.9 | 7.2 |
| Tebufenpyrad | 99 | 18.68 | 333.1>171.1 | 20 | 333.1>276.1 | 8 | 318.1>131.1 | 18 | 0.01 | 92 | 93 | 96 | 4.7 | 5.0 | 6.5 |
| Tefluthrin | 97 | 12.22 | 177.0>127.1 | 16 | 177.0>137.1 | 16 | 197.0>141.1 | 14 | 0.01 | 92 | 96 | 87 | 6.0 | 15.6 | 15.3 |
| Terbufos | 99 | 11.86 | 231.0>128.9 | 26 | 231.0>174.9 | 14 | 231.0>202.9 | 8 | 0.02 | 101 | 80 | 93 | 11.7 | 12.1 | 19.0 |
| Terbuthylazine | 99 | 11.89 | 229.1>173.1 | 6 | 214.1>71.0 | 16 | 214.1>132.1 | 8 | 0.01 | 97 | 84 | 89 | 12.3 | 16.3 | 18.7 |
| Tetrachlorvinphos | 99 | 15.34 | 330.9>109.0 | 22 | 328.9>109.0 | 20 | 333.0>109.0 | 15 | 0.01 | 102 | 79 | 98 | 8.6 | 9.7 | 11.9 |
| Tetradifon | 99 | 19.01 | 226.9>199.0 | 16 | 355.9>159.0 | 18 | 355.9>228.9 | 12 | 0.1 | 92 | 91 | 91 | 5.6 | 3.3 | 5.6 |
| Tetramethrin | 98 | 18.48 | 164.1>107.1 | 14 | 164.1>77.0 | 22 | 164.1>135.1 | 8 | 0.01 | 93 | 92 | 95 | 3.4 | 4.9 | 5.9 |
| Tolclofos-methyl | 98 | 13.15 | 264.9>249.9 | 14 | 264.9>93.0 | 24 | 264.9>219.9 | 22 | 0.01 | 94 | 75 | 99 | 8.4 | 11.4 | 17.9 |
| Tolylfluanid | 99 | 14.8 | 137.0>91.0 | 17 | 238>137.0 | 14 | 240.0>137.0 | 10 | 0.01 | 89 | 76 | 77 | 18.3 | 13.0 | 13.9 |
| trans-Chlordane | 99 | 15.28 | 374.8>265.9 | 26 | 372.8>263.9 | 28 | 372.8>265.9 | 22 | 0.01 | 80 | 82 | 92 | 15.0 | 14.4 | 10.7 |
| Transfluthrin | 99 | 13.17 | 163.1>143.1 | 16 | 163.1>127.1 | 6 | 163.1>91.0 | 12 | 0.01 | 92 | 84 | 86 | 14.2 | 13.1 | 17.7 |
| trans-Nonachlor | 99 | 15.56 | 406.8>299.9 | 24 | 406.8>334.9 | 16 | 406.8>109.0 | 22 | 0.01 | 79 | 78 | 93 | 18.5 | 11.8 | 12.4 |
| trans-Permethrine | 97 | 20.36 | 183.1>153.1 | 14 | 163.1>127.1 | 6 | 183.1>168.1 | 14 | 0.01 | 96 | 90 | 95 | 5.6 | 5.4 | 5.5 |
| Triadimefon | 99 | 14.16 | 208.1>181.0 | 10 | 208.1>111.0 | 22 | 208.1>127.0 | 14 | 0.01 | 94 | 93 | 84 | 11.2 | 15.5 | 19.0 |
| Triadimenol | 99 | 15.01 | 168.1>70.0 | 10 | 128.1>65.0 | 22 | 128.1>100.1 | 14 | 0.1 | 97 | 95 | 92 | 8.9 | 9.0 | 14.5 |
| Tri-allate | 99 | 12.36 | 268.1>184.0 | 20 | 270.1>186.0 | 20 | 268.1>226.0 | 14 | 0.01 | 90 | 79 | 87 | 16.6 | 9.9 | 19.4 |
| Triazophos | 97 | 17.11 | 161.0>134.0 | 8 | 161.0>106.0 | 14 | 257.0>162.0 | 8 | 0.01 | 88 | 90 | 96 | 8.2 | 9.5 | 7.5 |
| Trifloxystrobin | 99.6 | 17.26 | 222.1>190.1 | 4 | 222.1>130.1 | 12 | 222.1>162.1 | 10 | 0.1 | 92 | 93 | 95 | 8.7 | 5.5 | 7.7 |
| Triflumizole | 99 | 15.01 | 206.1>179.1 | 14 | 278.1>73.0 | 6 | 206.1>186.1 | 8 | 0.01 | 98 | 79 | 96 | 5.8 | 10.5 | 11.4 |
| Trifluralin | 98 | 10.54 | 306.1>264.1 | 8 | 264.1>160.1 | 18 | 264.1>206.1 | 8 | 0.01 | 86 | 90 | 83 | 16.3 | 17.4 | 13.9 |
| Vinclozolin | 99 | 13.06 | 212.0>172.0 | 16 | 285.0>212.0 | 12 | 212.0>145.0 | 24 | 0.01 | 94 | 90 | 90 | 13.1 | 15.0 | 15.8 |
