# Supplementary material for: Validation and Simultaneous Monitoring of 311 Pesticide Residues in Loamy Sand Agricultural Soils by LC-MS/MS and GC-MS/MS, Combined with QuEChERS-Based Extraction
Source: Molecules. 2023 May 23;28(11):4268. doi: 10.3390/molecules28114268 (PMC10254206; doi:10.3390/molecules28114268)
Supplement: Supplementary file 1 [file molecules-28-04268-s001.zip › Supplementary Table S2.docx]

**Table S2.** Data acquisition parameters and retention time for LC-MS/MS analysis. operated in multiple reaction monitoring (MRM) mode.

| **Analyte** | **Purity (%)** | **RT**  **(min)** | **Quantification Ion**  **transitions** | **CE**  **(eV)** | **Qualification Ion**  **transitions** | **CE**  **(eV)** | **LOQ** | **Average Recoveries**  **(n=6)** | | | | **RSD**  **(%)** | | | |
| --- | --- | --- | --- | --- | --- | --- | --- | --- | --- | --- | --- | --- | --- | --- | --- |
|  |  |  |  |  |  |  |  | **10**  **ppb** | **20**  **ppb** | **50**  **ppb** | **100**  **ppb** | **10**  **ppb** | **20**  **ppb** | **50**  **ppb** | **100**  **ppb** |
| 3-hydroxycarbofuran | 96 | 10.78 | 255.0>163.2 | -19 | 255.0>220.1 | -11 | 0.01 | 90.1 | 82.7 | 87.5 | 86.6 | 14.2 | 17.8 | 8.5 | 4.8 |
| Acetamiprid | 99 | 10.69 | 223.1>126.1 | -21 | 223.1>56.0 | -16 | 0.01 | 106 | 91.8 | 89.2 | 92.2 | 13.1 | 8.96 | 4.07 | 3.27 |
| Aldicarb | 98 | 13.13 | 208.2>115.9 | -9 | 208.2>89.0 | -18 | 0.01 | 91.2 | 90.6 | 84.9 | 92.9 | 4.46 | 8.47 | 12.4 | 6.92 |
| Aldicarb sulfone | 99 | 3.51 | 240.1>86.2 | -21 | 240.1>148.2 | -14 | 0.01 | 95.4 | 92.6 | 91.3 | 95.8 | 4.43 | 7.88 | 1.55 | 2.83 |
| Aldicarb sulfoxide | 99 | 2.93 | 228.8>166.1 | -11 | 228.8>109.1 | -17 | 0.01 | 91 | 88.9 | 87.8 | 90.9 | 10.2 | 8.44 | 5.88 | 1.59 |
| Ametryn | 99 | 17.97 | 228.1>186.0 | -19 | 228.1>68.0 | -40 | 0.01 | 104 | 93.1 | 90.9 | 98.2 | 7.2 | 7.65 | 5.48 | 4.56 |
| Amitraz | 99 | 29.85 | 294.1>163.0 | -15 | 294.1>122.2 | -34 | 0.01 | 79.6 | 89.5 | 87.9 | 76.3 | 7.58 | 8.01 | 1.32 | 1.93 |
| Azoxystrobin | 99 | 19.14 | 404.0>372.0 | -15 | 404.0>329.0 | -31 | 0.01 | 104 | 88.8 | 86.2 | 93.9 | 9.16 | 12.4 | 3.26 | 4.38 |
| Benalaxyl | 99 | 23.61 | 326.2>148.1 | -21 | 326.2>91.1 | -37 | 0.01 | 90.8 | 92.1 | 93.3 | 95.9 | 11.04 | 5 | 6.28 | 3.88 |
| Bendiocarb | 99 | 15.18 | 224.1>167.0 | -10 | 224.1>109.0 | -19 | 0.01 | 93.8 | 102 | 95.5 | 96.6 | 13.2 | 7.22 | 5.56 | 5.34 |
| Benzoximate | 99 | 24.53 | 364.1>199.0 | -10 | 364.1>77.1 | -52 | 0.01 | 92.9 | 94.2 | 88.7 | 92.4 | 9.38 | 5.74 | 3.63 | 1.77 |
| Boscalid | 99 | 20.21 | 343.0>307.0 | -21 | 343.0>271.0 | -31 | 0.01 | 96 | 95.2 | 89.25 | 95.23 | 9.95 | 5.37 | 3.86 | 4.37 |
| Bromuconazole | 99 | 22.62 | 377.9>159.1 | -26 | 377.9>160.9 | -29 | 0.01 | 86.5 | 98.3 | 93.8 | 91.6 | 5.33 | 4.84 | 5.43 | 5.29 |
| Bupirimate | 99 | 21.74 | 317.2>166.0 | -24 | 317.2>108.0 | -26 | 0.01 | 97.3 | 86.8 | 94.3 | 94.4 | 13.5 | 3.8 | 8.8 | 3.3 |
| Buprofezin | 99 | 26.76 | 306.2>201.1 | -12 | 306.2>57.0 | -24 | 0.01 | 93.8 | 97.6 | 92.4 | 93.3 | 7.86 | 5.03 | 3.48 | 2.11 |
| Butafenacil | 99 | 21.47 | 492.1>330.9 | -24 | 492.1>179.9 | -42 | 0.01 | 97.1 | 89 | 94.6 | 92.73 | 9.61 | 8.67 | 4.71 | 4.64 |
| Butocarboxim | 99 | 12.90 | 213.1>75.1 | -19 | 213.1>116.0 | -17 | 0.02 | 95.4 | 93.9 | 95.9 | 109 | 12.4 | 15.4 | 8.4 | 13.9 |
| Butoxycarboxim | 99 | 3.26 | 245.1>130.1 | -16 | 245.1>103.0 | -15 | 0.01 | 95.1 | 113 | 91.9 | 93.7 | 4 | 9.58 | 4.79 | 1.38 |
| Carbaryl | 99 | 15.89 | 202.1>145.1 | -12 | 202.1>127.0 | -25 | 0.01 | 101 | 87.3 | 79.5 | 88.8 | 12.6 | 10.9 | 8.19 | 5.51 |
| Carbendazim | 99 | 5.89 | 192.1>160.2 | -19 | 192.1>132.2 | -30 | 0.01 | 95.4 | 81.3 | 87.8 | 87.5 | 4.6 | 7.8 | 2.47 | 0.83 |
| Carbetamide | 94 | 14.19 | 237.1>192.1 | -9 | 237.1>118.2 | -12 | 0.01 | 92.9 | 88.3 | 94. | 90.8 | 12.6 | 12.7 | 12.3 | 4.11 |
| Carbofuran | 99 | 15.27 | 222.1>123.2 | -19 | 222.1>165.0 | -11 | 0.01 | 111 | 111 | 102.7 | 108.7 | 5.19 | 6.83 | 9.99 | 4.71 |
| Carboxin | 99 | 15.83 | 236.1>143.1 | -15 | 236.1>43.0 | -36 | 0.01 | 93.1 | 92.35 | 88.06 | 89.35 | 5.72 | 6.36 | 3.91 | 2.84 |
| Chlorantraniliprole | 99 | 18.55 | 483.9>452.9 | -19 | 483.9>285.9 | -17 | 0.01 | 92.5 | 89.6 | 90.6 | 97.4 | 6.32 | 6.65 | 2.8 | 1.53 |
| Chlorfluazuron | 99 | 29.43 | 539.9>382.9 | -20 | 539.9>158.0 | -21 | 0.01 | 95.4 | 94 | 88.9 | 93.4 | 15.28 | 10.3 | 7.96 | 4.77 |
| Chlorotoluron | 99 | 16.69 | 213.1>72.2 | -22 | 213.1>46.1 | -15 | 0.01 | 80.3 | 92.6 | 95.3 | 89.9 | 12.74 | 5.96 | 13.02 | 2.2 |
| Chloroxuron | 99 | 20.83 | 291.1>72.2 | -23 | 291.1>46.2 | -22 | 0.01 | 97.9 | 88.7 | 90.6 | 97.7 | 12.64 | 4.54 | 10.79 | 4.21 |
| Clethodim isomer II | 95 | 26.14 | 360.1>164.2 | -21 | 360.1>166.1 | -27 | 0.01 | 82.7 | 76.2 | 74.9 | 82.0 | 11.5 | 12.1 | 13.32 | 2.55 |
| Clofentezine | 99 | 24.38 | 303.0>138.2 | -15 | 303.0>102.1 | -37 | 0.01 | 102 | 96.4 | 92.9 | 93.9 | 9.55 | 10.7 | 5.38 | 8.75 |
| Clothianidin | 99 | 8.95 | 250.0>169.1 | -13 | 250.0>132.1 | -16 | 0.01 | 86.7 | 82.3 | 80.2 | 92.3 | 13.52 | 8.05 | 4.11 | 4.03 |
| Cycluron | 99 | 17.86 | 199.2>89.0 | -15 | 199.2>72.2 | -24 | 0.01 | 100 | 95 | 88.4 | 96.2 | 5.91 | 7.16 | 3.63 | 1.81 |
| Cyproconazole | 97 | 21.07 | 292.1>70.1 | -22 | 292.1>125.1 | -32 | 0.01 | 96.9 | 94.5 | 93.8 | 94.6 | 6.91 | 5.67 | 5.49 | 7.48 |
| Cyprodinil | 99 | 22.45 | 226.2>93.1 | -37 | 226.2>77.1 | -27 | 0.01 | 96.8 | 107 | 90.1 | 94.1 | 5.92 | 5.88 | 1.71 | 0.8 |
| Diclobutrazol | 99 | 22.96 | 328.0>70.1 | -22 | 328.0>159.0 | -35 | 0.01 | 90.8 | 105 | 89.2 | 97.3 | 13.6 | 2.46 | 7.68 | 5.2 |
| Dicrotophos | 96 | 8.63 | 237.9>112.2 | -13 | 237.9>72.0 | -26 | 0.01 | 87.7 | 89.9 | 104 | 97.6 | 10.1 | 7.75 | 7.5 | 3.74 |
| Diethofencarb | 99 | 19.27 | 268.2>226.1 | -10 | 268.2>124.2 | -31 | 0.01 | 95 | 99.1 | 95.7 | 99.2 | 13.9 | 12.7 | 11.3 | 4.6 |
| Difenoconazole | 99 | 25.02 | 406.1>250.9 | -25 | 406.1>111.0 | -55 | 0.01 | 101 | 96.0 | 92.6 | 94.5 | 5.05 | 5.73 | 4.93 | 2.15 |
| Diflubenzuron | 99 | 22.19 | 311.1>158.1 | -16 | 311.1>141.1 | -32 | 0.01 | 96.8 | 103 | 90.6 | 93.2 | 6.75 | 5.82 | 4.47 | 1.8 |
| Dimethoate | 99 | 10.26 | 230.0>198.9 | -10 | 230.0>125.0 | -21 | 0.01 | 89.8 | 91.7 | 93.3 | 97.7 | 9.92 | 11.8 | 3.08 | 4.26 |
| Dimethomorph | 99 | 20.19 | 388.1>301.0 | -21 | 388.1>165.1 | -34 | 0.01 | 64.6 | 83.3 | 79.5 | 89.5 | 2.33 | 1.56 | 3.43 | 1.31 |
| Dimoxystrobin | 99 | 22.82 | 327.1>116.1 | -22 | 327.1>205.0 | -11 | 0.01 | 97.4 | 98.9 | 87.7 | 91.5 | 9.79 | 4.46 | 4.06 | 2.1 |
| Dioxacarb | 99 | 10.51 | 224.1>123.0 | -16 | 224.1>167.0 | -9 | 0.01 | 95.4 | 91.7 | 88.4 | 94.7 | 12.9 | 8.53 | 7 | 4.08 |
| Diuron | 99 | 17.56 | 234.8>72.1 | -23 | 234.8>46.1 | -16 | 0.01 | 107 | 85.8 | 101 | 99.6 | 10.7 | 9.79 | 3.69 | 7.31 |
| Emamectin-benzoate b1a | 99 | 27.06 | 886.4>158.2 | -36 | 886.4>82.1 | -55 | 0.01 | 101 | 95.1 | 88.2 | 90.5 | 6.26 | 4.94 | 3.59 | 3.63 |
| Epoxiconazole | 99 | 21.79 | 330.0>121.1 | -21 | 330.0>101.1 | -44 | 0.01 | 93.9 | 95.3 | 97.9 | 92.9 | 8.15 | 11.4 | 3.84 | 1.79 |
| Etaconazole | 97 | 21.58 | 328.1>159.0 | -28 | 328.1>55.0 | -22 | 0.01 | 109 | 100 | 95.2 | 99.2 | 11.4 | 8.02 | 15.9 | 5.73 |
| Ethiprole | 99 | 19.63 | 397.0>350.9 | -22 | 397.0>254.9 | -36 | 0.02 | 88.5 | 89.5 | 92.2 | 94.4 | 7.5 | 5.6 | 10.3 | 10.7 |
| Ethirimol | 97 | 13.51 | 210.2>140.2 | -21 | 210.2>98.2 | -27 | 0.02 | 80.3 | 85.1 | 79.4 | 72.6 | 8.3 | 9.1 | 12 | 4.1 |
| Ethofumesate | 99 | 19.29 | 304.1>287.0 | -11 | 304.1>121.1 | -22 | 0.02 | 87.4 | 96.5 | 89.8 | 90.7 | 11.8 | 18.4 | 13.1 | 6.81 |
| Etoxazole | 97 | 28.64 | 360.1>141.1 | -30 | 360.1>113.1 | -55 | 0.01 | 93.6 | 96.2 | 91.2 | 96.1 | 3.25 | 2.44 | 2.44 | 1.28 |
| Fenamidone | 99 | 19.67 | 312.1>92.1 | -26 | 312.1>236.0 | -15 | 0.01 | 90 | 95.4 | 89.2 | 96 | 7.46 | 10.9 | 6.21 | 6.98 |
| Fenarimol | 99 | 21.38 | 331.0>268.0 | -23 | 331.0>110.9 | -55 | 0.01 | 93.4 | 81.4 | 82.3 | 99.7 | 13.5 | 15.8 | 12.3 | 13.5 |
| Fenazaquin | 99 | 30.21 | 307.2>57.0 | -23 | 307.2>161.1 | -17 | 0.01 | 96.2 | 100 | 90 | 94.3 | 2.92 | 3.69 | 3.25 | 2.17 |
| Fenbuconazole | 99 | 22.01 | 337.1>125.1 | -29 | 337.1>70.1 | -22 | 0.01 | 97.1 | 85.4 | 85.4 | 92.9 | 13.7 | 13.5 | 7.8 | 6.07 |
| Fenhexamid | 99 | 21.358 | 304.1>97.1 | -24 | 304.1>55.0 | -40 | 0.01 | 82.7 | 69.63 | 83.6 | 79.1 | 14 | 16.2 | 11.2 | 13.9 |
| Fenoxycarb | 99 | 22.537 | 302.1>88.0 | -22 | 302.1>116.2 | -11 | 0.02 | 87.9 | 94.35 | 84 | 98.9 | 8.3 | 7.67 | 10.2 | 6.72 |
| Fenpropimorph | 95 | 18.893 | 304.2>147.1 | -29 | 304.2>117.0 | -55 | 0.01 | 96.5 | 105 | 88.5 | 91.3 | 14.1 | 9.17 | 2.26 | 4.96 |
| Fenpyroximate | 99 | 29.281 | 422.1>366.0 | -17 | 422.1>138.1 | -32 | 0.01 | 97.7 | 94.3 | 91.3 | 94.4 | 4.4 | 3.98 | 4.69 | 2.22 |
| Fenuron | 99 | 9.589 | 165.0>72.2 | -22 | 165.0>46.1 | -14 | 0.01 | 93.2 | 90.2 | 92.2 | 94.5 | 9.8 | 6.52 | 4.58 | 2.89 |
| Fipronil | 97 | 22.464 | 435.0>330.0 | 16 | 435.0>250.1 | 27 | 0.01 | 90.9 | 98.4 | 94.6 | 89.2 | 14.7 | 16.7 | 10.5 | 3.98 |
| Flonicamid | 99 | 5.193 | 230.1>203.0 | -16 | 230.1>148.1 | -26 | 0.01 | 104 | 95.6 | 94.8 | 96.3 | 10.7 | 9.77 | 5.57 | 2.41 |
| Fluazinam | 99 | 27.302 | 463.0>416.0 | 20 | 463.0>398.0 | 17 | 0.01 | 96.9 | 89.2 | 91.9 | 94.6 | 6.21 | 7.66 | 4.15 | 2.02 |
| Fludioxonil | 99 | 19.872 | 247.0>180.2 | 28 | 247.0>126.2 | 29 | 0.01 | 100 | 91.6 | 96.5 | 93.3 | 1.86 | 3.1 | 2.3 | 1.81 |
| Flufenacet | 98 | 21.666 | 364.1>152.1 | -19 | 364.1>194.0 | -12 | 0.01 | 95.5 | 98.8 | 93.3 | 94.8 | 8.24 | 6.85 | 8.38 | 3.03 |
| Flufenoxuron | 98 | 28.535 | 489.1>158.1 | -21 | 489.1>140.9 | -46 | 0.01 | 91.5 | 89.5 | 92.6 | 96.2 | 6.1 | 9.01 | 5.38 | 2.56 |
| Fluometuron | 99 | 16.479 | 233.1>72.1 | -22 | 233.1>46.2 | -18 | 0.01 | 90.7 | 109.1 | 92.3 | 93.7 | 6.35 | 3.96 | 5.63 | 3.31 |
| Fluoxastrobin | 99 | 21.294 | 458.8>427.0 | -17 | 458.8>188.1 | -35 | 0.01 | 92.1 | 97.4 | 91.5 | 91.5 | 17.8 | 15.57 | 4.3 | 9.25 |
| Fluquinconazole | 99 | 21.21 | 376.0>349.0 | -20 | 376.0>108.0 | -51 | 0.01 | 96 | 103 | 96.7 | 97 | 9.59 | 11.3 | 5.78 | 3.79 |
| Flusilazole | 98 | 22.38 | 316.1>247.0 | -18 | 316.1>165.1 | -26 | 0.01 | 92.91 | 90.1 | 87.9 | 96.7 | 7.18 | 7.27 | 2.04 | 9.85 |
| Flutolanil | 99 | 20.21 | 324.1>242.0 | -25 | 324.1>261.9 | -19 | 0.01 | 102.4 | 99.8 | 90.8 | 96.8 | 2.27 | 4.02 | 7.66 | 5.06 |
| Flutriafol | 98 | 17.29 | 302.1>70.1 | -22 | 302.1>123.0 | -26 | 0.01 | 93.1 | 97.4 | 91.9 | 92.5 | 12.2 | 7.83 | 8.28 | 7.26 |
| Forchlorfenuron | 99 | 17.61 | 248.1>129.2 | -17 | 248.1>93.2 | -34 | 0.01 | 91.6 | 92.7 | 82.2 | 86.1 | 16.4 | 13.4 | 10.9 | 7.27 |
| formetanate HCl | 85 | 2.12 | 222.2>165.2 | -15 | 222.2>93.1 | -37 | 0.01 | 79.6 | 79.3 | 75.5 | 77.3 | 2 | 1.21 | 1.46 | 0.97 |
| Furalaxyl | 99 | 19.23 | 302.1>242.1 | -16 | 302.1>95.0 | -27 | 0.01 | 95.3 | 88.1 | 89.5 | 93.1 | 6.19 | 9.11 | 4.76 | 4.27 |
| Furathiocarb | 97 | 26.48 | 383.2>195.0 | -36 | 383.2>252.0 | -55 | 0.01 | 93.8 | 94.2 | 89.4 | 96.1 | 6.08 | 3.79 | 4.67 | 1.51 |
| Hexaconazole | 99 | 23.89 | 314.7 >70.0 | -22 | 314.1>159.0 | -30 | 0.01 | 93.8 | 95.7 | 91.4 | 92.1 | 13.7 | 6.44 | 9.43 | 6.87 |
| Hexythiazox | 99 | 27.94 | 353.1>228.0 | -16 | 353.1>168.0 | -24 | 0.01 | 101 | 95.1 | 90.9 | 95.2 | 3.68 | 11.4 | 3.32 | 3.2 |
| Hydramethylnon | 99 | 24.74 | 495.1>323.0 | -31 | 495.1>151.0 | -55 | 0.01 | 87 | 83.6 | 77.3 | 88.2 | 6.68 | 7.07 | 2.57 | 2.29 |
| Imazalil | 98 | 16.54 | 297.1>159.1 | -23 | 297.1>41.1 | -31 | 0.01 | 95.9 | 87.9 | 80.2 | 91.2 | 7.69 | 14.3 | 16.7 | 10.9 |
| Imidacloprid | 99 | 8.96 | 256.1>209.0 | -17 | 256.1>175.0 | -20 | 0.01 | 76.9 | 88.6 | 89.4 | 91 | 6.47 | 1.68 | 3.54 | 1.5 |
| Indoxacarb | 99 | 25.42 | 528.1>203.0 | -37 | 528.1>249.1 | -18 | 0.01 | 103 | 101 | 83.2 | 95.9 | 12.1 | 10.3 | 14.9 | 5.02 |
| Ipconazole | 99 | 25.64 | 334.1>70.1 | -22 | 334.1>125.1 | -37 | 0.01 | 95.5 | 94.9 | 93.8 | 95.9 | 3.54 | 2.67 | 2.98 | 1.26 |
| Iprovalicarb | 99 | 21.44 | 321.2>119.2 | -21 | 321.2>91.0 | -52 | 0.01 | 99 | 96.6 | 93.5 | 98.1 | 11.1 | 9.85 | 4.65 | 2.52 |
| Isoproturon | 99 | 17.48 | 207.2>72.2 | -23 | 207.2>46.1 | -17 | 0.01 | 95.8 | 97.9 | 90.4 | 98.1 | 8.8 | 6.41 | 6.15 | 1.73 |
| kresoxim methyl | 99 | 23.09 | 314.1>267.0 | -8 | 314.1>222.0 | -16 | 0.01 | 92.3 | 96.1 | 92.1 | 95.7 | 11.6 | 5.39 | 3.87 | 3.17 |
| Linuron | 99 | 19.05 | 248.8>160.0 | -18 | 248.8>182.1 | -16 | 0.01 | 95.6 | 85.3 | 88.9 | 99 | 15.1 | 16.4 | 7.79 | 3.61 |
| Lufenuron | 99 | 27.36 | 508.9>339.0 | 12 | 508.9>326.0 | 19 | 0.01 | 97.4 | 97.4 | 95.6 | 88.2 | 17.7 | 4.73 | 7.89 | 4.93 |
| Mandipropamid | 99 | 19.41 | 412.1>327.9 | -15 | 412.1>125.1 | -36 | 0.01 | 106 | 103 | 95.8 | 90.2 | 8.63 | 14.3 | 10.6 | 5.65 |
| Mefenacet | 99 | 20.84 | 299.0>148.2 | -14 | 299.0>120.2 | -24 | 0.01 | 102.2 | 90 | 92.8 | 93.9 | 5.22 | 7.16 | 6.24 | 3.99 |
| Mepronil | 99 | 20.39 | 270.2>119.1 | -23 | 270.2>91.1 | -41 | 0.01 | 89.5 | 95.1 | 91.2 | 98.3 | 10.4 | 6.8 | 10.1 | 3.38 |
| Metaflumizone | 99 | 27.76 | 507.1>178.1 | -27 | 507.1>287.0 | -25 | 0.01 | 85.6 | 82.5 | 92.4 | 93.1 | 10.5 | 8.2 | 6.89 | 7.95 |
| Metalaxyl | 99 | 17.69 | 280.1>220.0 | -14 | 280.1>192.1 | -18 | 0.01 | 89.2 | 96.8 | 92.6 | 92.5 | 11.1 | 12.8 | 8.27 | 3.55 |
| Metconazole | 99 | 24.09 | 320.1>70.2 | -23 | 320.1>125.1 | -35 | 0.01 | 88.1 | 91.9 | 90.4 | 92.9 | 14.1 | 3.65 | 4.23 | 3.61 |
| Methabenzthiazuron | 99 | 17.09 | 222.1>165.1 | -17 | 222.1>150.1 | -32 | 0.01 | 107 | 82.3 | 85.9 | 95.6 | 12.2 | 10.4 | 8.36 | 4.22 |
| Methamidophos | 99 | 2.08 | 142.2>94.0 | -15 | 142.2>124.9 | -16 | 0.01 | 79.3 | 101 | 74.9 | 73.5 | 4.6 | 8.81 | 3.38 | 2.44 |
| Methiocarb | 99 | 19.41 | 226.1>169.1 | -10 | 226.1>121.1 | -19 | 0.01 | 97.6 | 87.9 | 89.5 | 87.1 | 10.5 | 16.4 | 7.11 | 9.36 |
| Methomyl | 99 | 4.81 | 163.1>88.1 | -13 | 163.1>106.0 | -13 | 0.01 | 110 | 117 | 114 | 118 | 7.39 | 7.6 | 4.02 | 1.3 |
| Methoprotryne | 98 | 18.24 | 272.2>198.0 | -19 | 272.2>170.0 | -17 | 0.01 | 93.7 | 94.5 | 88.1 | 95.8 | 6.99 | 7.81 | 6.54 | 3.29 |
| Methoxyfenozide | 99 | 20.51 | 369.2>149.2 | -18 | 369.2>313.0 | -9 | 0.01 | 90.3 | 96.2 | 89.6 | 96.9 | 11.8 | 11.2 | 8.18 | 8.13 |
| Metobromuron | 99 | 16.87 | 259.1>170.0 | -19 | 259.1>148.1 | -15 | 0.02 | 93.4 | 92.2 | 92.1 | 97.3 | 13.8 | 15.7 | 12.2 | 6.63 |
| Metribuzin | 98 | 14.92 | 215.1>187.1 | -18 | 215.1>49.1 | -26 | 0.02 | 84.6 | 93.4 | 105 | 86.3 | 12.9 | 13.6 | 13.3 | 13.7 |
| Mevinphos | 98 | 10.77 | 225.1>127.0 | -17 | 225.1>193.0 | -9 | 0.01 | 98.1 | 92.7 | 93.4 | 91.6 | 10.2 | 8.63 | 6.05 | 8.15 |
| Mexacarbate | 98 | 11.49 | 223.1>151.2 | -24 | 223.1>166.1 | -14 | 0.01 | 83.3 | 80.9 | 85.6 | 86.6 | 13.5 | 9.72 | 0.98 | 3.18 |
| Monocrotophos | 96 | 8.07 | 240.9>127.1 | -21 | 240.9>224.0 | -7 | 0.01 | 92.8 | 95.1 | 87.7 | 92.7 | 5.68 | 9.6 | 4.07 | 2.01 |
| Monolinuron | 99 | 16.18 | 215.1>126.1 | -18 | 215.1>99.1 | -34 | 0.02 | 96.3 | 83.21 | 94.6 | 95.5 | 14.7 | 16.72 | 8.33 | 12.2 |
| Myclobutanil | 99 | 20.54 | 289.1>70.1 | -22 | 289.1>125.0 | -31 | 0.01 | 77.74 | 90.4 | 97.4 | 79.8 | 13.1 | 8.42 | 8.03 | 11.8 |
| Neburon | 99 | 22.62 | 274.8>88.0 | -17 | 274.8>57.0 | -23 | 0.01 | 87.62 | 100 | 95.2 | 89.93 | 12 | 10.7 | 8.67 | 5.49 |
| Nitenpyram | 98 | 3.933 | 271.1>225.0 | -12 | 271.1>126.1 | -30 | 0.01 | 96.88 | 88.2 | 78.9 | 87.01 | 4.62 | 4.69 | 3.13 | 3.3 |
| Nuarimol | 99 | 19.31 | 315.1>252.0 | -22 | 315.1>81.1 | -29 | 0.02 | 82.4 | 109 | 77.9 | 100.33 | 16.4 | 10.9 | 15.6 | 8.44 |
| Omethoate | 97 | 2.67 | 214.1>125.0 | -23 | 214.1>183.0 | -11 | 0.01 | 89.91 | 110 | 86.2 | 89.31 | 3.43 | 6.18 | 2.52 | 1.41 |
| Oxadixyl | 99 | 14.1 | 296.2>279.1 | -8 | 296.2>219.1 | -15 | 0.01 | 89.56 | 100 | 99.1 | 94.12 | 17.6 | 14.5 | 16.1 | 13.1 |
| Oxamyl | 99 | 3.78 | 237.1>72.1 | -22 | 237.1>90.0 | -8 | 0.01 | 96.44 | 99.2 | 91.1 | 94.25 | 4.62 | 4.07 | 3.04 | 3.92 |
| Paclobutrazol | 99 | 19.94 | 294.1>70.1 | -22 | 294.1>125.1 | -36 | 0.01 | 94.07 | 89.2 | 91 | 97.74 | 10.2 | 13.2 | 8.54 | 6.19 |
| Penconazole | 99 | 22.99 | 284.1>70.1 | -17 | 284.1>159.0 | -30 | 0.01 | 96.12 | 101 | 88.5 | 95.22 | 14.1 | 7.16 | 10.9 | 5.2 |
| Pencycuron | 99 | 24.73 | 329.1>125.0 | -25 | 329.1>89.0 | -55 | 0.01 | 95.32 | 95.5 | 91.3 | 95.03 | 6.96 | 4.44 | 1.43 | 1.01 |
| Picoxystrobin | 99 | 22.59 | 368.0>145.1 | -22 | 368.0>205.0 | -10 | 0.01 | 99.33 | 94.4 | 92.7 | 94.26 | 11.8 | 7.86 | 3.6 | 2.93 |
| Piperonyl butoxide | 95 | 27.19 | 356.2>177.0 | -14 | 356.2>119.0 | -33 | 0.01 | 97.53 | 94 | 88.6 | 94.12 | 4.22 | 2.64 | 2.52 | 2.61 |
| Pirimicarb | 98 | 14.58 | 239.2>72.0 | -23 | 239.2>182.1 | -16 | 0.01 | 91.07 | 97.8 | 81.1 | 94.17 | 11.3 | 5.77 | 3.11 | 7.98 |
| Prochloraz | 99 | 23.9 | 376.0>308.0 | -13 | 376.0>70.0 | -26 | 0.01 | 98.76 | 100 | 93 | 99.29 | 9.73 | 9.05 | 9.74 | 4.78 |
| Promecarb | 99 | 20.07 | 208.1>109.1 | -15 | 208.1>151.2 | -10 | 0.01 | 92.26 | 106 | 86.7 | 99.18 | 10.4 | 7.22 | 13.6 | 7.61 |
| Prometon | 97 | 16.76 | 226.2>142.0 | -22 | 226.2>184.1 | -17 | 0.01 | 99.78 | 96.3 | 93.4 | 93.63 | 9.54 | 3.54 | 6.42 | 4.62 |
| Prometryn | 99 | 20.19 | 242.1>158.0 | -22 | 242.1>200.2 | -19 | 0.01 | 91.82 | 91.5 | 89.3 | 99.89 | 10.5 | 5.71 | 3.91 | 3.18 |
| Propargite | 99 | 28.72 | 368.2>231.1 | -11 | 368.2>175.1 | -16 | 0.01 | 88.21 | 96.6 | 93.8 | 94.55 | 9.36 | 7.27 | 4.88 | 2.56 |
| Propham | 99 | 17.04 | 180.1>138.0 | -10 | 180.1>120.2 | -15 | 0.02 | 93.7 | 102.7 | 89.9 | 92.25 | 12.6 | 11.7 | 14.1 | 11.7 |
| Propiconazole | 98 | 23.63 | 342.0>158.9 | -28 | 342.0>69.1 | -22 | 0.01 | 106 | 93.8 | 95.1 | 95.52 | 6.8 | 6.12 | 8.75 | 6.68 |
| Propoxur | 99 | 15.14 | 209.9>111.2 | -14 | 209.9>93.1 | -24 | 0.01 | 80.9 | 103 | 97.2 | 98.33 | 13.7 | 6.17 | 8.96 | 8.91 |
| Pyraclostrobin | 99 | 24.14 | 388.1>194.1 | -13 | 388.1>163.1 | -24 | 0.01 | 74.9 | 88 | 92.6 | 91.77 | 3.15 | 0.95 | 1.7 | 0.98 |
| Pyridaben | 99 | 30.21 | 365.2>147.1 | -25 | 365.2>308.9 | -13 | 0.01 | 94.8 | 96.9 | 91.4 | 94.52 | 4.37 | 4.94 | 2.08 | 1.83 |
| Pyrimethanil | 99 | 18.67 | 200.1>107.0 | -24 | 200.1>82.0 | -27 | 0.01 | 96.8 | 101 | 94.5 | 94.87 | 13.1 | 9.97 | 4.07 | 4.65 |
| Pyriproxyfen | 99 | 27.67 | 322.1>96.1 | -16 | 322.1>78.1 | -54 | 0.01 | 97.2 | 90.4 | 91.6 | 95.76 | 5.42 | 8.77 | 2.99 | 2.77 |
| Quinoxyfen | 99 | 27.48 | 308.0>197.0 | -31 | 308.0>162.0 | -46 | 0.01 | 94.8 | 89.7 | 87.7 | 93.38 | 11.4 | 11.4 | 3.77 | 5.06 |
| Rotenone | 98 | 22.33 | 395.1>213.0 | -23 | 395.1>192.0 | -24 | 0.01 | 109 | 96.3 | 91.6 | 101 | 9.78 | 14.1 | 15.9 | 9.96 |
| Secbumeton | 96 | 16.76 | 226.2>142.1 | -22 | 226.2>100.2 | -28 | 0.01 | 96.3 | 97 | 90.1 | 95.97 | 5.69 | 4.4 | 6.45 | 4.32 |
| Simetryn | 99 | 15.75 | 214.1>68.0 | -35 | 214.1>124.0 | -21 | 0.01 | 93.9 | 90.7 | 85.5 | 94.74 | 10.9 | 9.2 | 9.85 | 4.61 |
| Spinetoram | 97 | 25.23 | 748.5>142.2 | -31 | 748.5>98.1 | -55 | 0.01 | 93.8 | 91.3 | 93 | 89.93 | 12.6 | 4.3 | 6.11 | 3.09 |
| Spinosyn A | 81 | 23.86 | 732.6>142.2 | -29 | 732.6>98.1 | -53 | 0.01 | 95.3 | 92.6 | 97.6 | 91.81 | 7.35 | 7.7 | 4.11 | 4.14 |
| Spinosyn D | 19 | 24.99 | 746.6>142.1 | -30 | 746.6>98.1 | -54 | 0.01 | 101 | 93.9 | 90.2 | 93.38 | 5.89 | 3.7 | 3.38 | 4.9 |
| Spirodiclofen | 99 | 29.39 | 411.1>71.1 | -22 | 411.1>313.1 | -14 | 0.01 | 90.2 | 93.9 | 93.6 | 97.17 | 4.19 | 6.8 | 4.02 | 5.74 |
| Spirotetramat | 99 | 21.46 | 374.1>330.1 | -15 | 374.1>216.0 | -33 | 0.01 | 93.6 | 94.5 | 90.99 | 95.78 | 14.6 | 12.5 | 6.82 | 2.84 |
| Spiroxamine | 98 | 19.07 | 298.2>144.2 | -20 | 298.2>100.2 | -30 | 0.01 | 87.4 | 88.5 | 86.81 | 87.48 | 6.61 | 6.4 | 6.01 | 6.32 |
| Tebuconazole | 99 | 23.27 | 308.2>70.1 | -22 | 308.2>125.1 | -38 | 0.01 | 91.5 | 98.4 | 88.63 | 93.12 | 5.64 | 3.01 | 6.55 | 2.88 |
| Tebufenozide | 99 | 22.58 | 353.2>133.1 | -21 | 353.2>297.0 | -9 | 0.01 | 85.8 | 93.8 | 91 | 94.69 | 10.9 | 12.1 | 8.69 | 3.23 |
| Tebufenpyrad | 99 | 26.93 | 334.2>117.0 | -35 | 334.2>145.2 | -27 | 0.01 | 93.6 | 92.9 | 87.2 | 94.53 | 4.41 | 6.12 | 4.14 | 2.04 |
| Tebuthiuron | 99 | 15.60 | 229.1>172.0 | -17 | 229.1>116.0 | -27 | 0.01 | 106.2 | 94.8 | 93.9 | 92.7 | 7.14 | 7.86 | 8.04 | 10.5 |
| Temephos | 94 | 26.89 | 467.0>125.0 | -38 | 467.0>418.8 | -19 | 0.01 | 93.7 | 97.5 | 93 | 97.6 | 4.84 | 11.4 | 6.39 | 3.81 |
| Terbutryn | 98 | 20.46 | 242.1>186.0 | -19 | 242.1>96.1 | -31 | 0.01 | 93.5 | 94.1 | 92.3 | 95.4 | 4.84 | 9.23 | 2.24 | 2.81 |
| Tetraconazole | 97 | 21.58 | 372.0>159.0 | -30 | 372.0>70.1 | -22 | 0.02 | 93.9 | 89.9 | 94.5 | 89.8 | 9.53 | 8.75 | 7.86 | 4.1 |
| Thiabendazole | 99 | 8.43 | 202.0>175.0 | -25 | 202.0>131.2 | -32 | 0.01 | 74.6 | 93.5 | 73.6 | 75.4 | 9.18 | 11.1 | 4.47 | 3.53 |
| Thiacloprid | 99 | 12.1 | 253.0>126.1 | -21 | 253.0>90.1 | -39 | 0.01 | 86.2 | 103 | 99.6 | 96.4 | 7.54 | 8.54 | 6.16 | 7.24 |
| Thiamethoxam | 99 | 5.36 | 292.0>211.1 | -13 | 292.0>181.1 | -22 | 0.01 | 83.9 | 101 | 91.2 | 94.3 | 7.82 | 7.32 | 5.5 | 3.58 |
| Thidiazuron | 99 | 15.04 | 221.0>102.0 | -16 | 221.0>128.0 | -16 | 0.01 | 80.5 | 93.8 | 73.5 | 85.1 | 11.7 | 10.4 | 8.41 | 3.97 |
| Thiobencarb | 98 | 24.64 | 257.8>125.1 | -21 | 257.8>89.0 | -50 | 0.01 | 87.3 | 88.4 | 89.4 | 98.4 | 8.92 | 5.35 | 7.83 | 2.89 |
| Thiophanate methyl | 99 | 15.1 | 343.0>151.2 | -21 | 343.0>93.0 | -50 | 0.02 | 89.6 | 93.6 | 83.4 | 88 | 14.7 | 5.2 | 18.7 | 14.6 |
| Triadimefon | 99 | 20.62 | 294.1>197.0 | -15 | 294.1>69.0 | -22 | 0.02 | 94.1 | 105 | 88.4 | 100 | 11.5 | 16.6 | 12.5 | 9.93 |
| Triadimenol | 99 | 21.05 | 296.1>70.1 | -22 | 298.1>70.1 | -22 | 0.01 | 96.1 | 97.2 | 93.2 | 94.4 | 8.73 | 6.9 | 4.66 | 3.45 |
| Trichlorfon | 99 | 12.57 | 256.9>127.0 | -23 | 256.9>109.1 | -25 | 0.01 | 93.8 | 79.6 | 94.2 | 94.8 | 3.78 | 13.2 | 9.29 | 4.46 |
| Tricyclazole | 99 | 12.79 | 190.1>136.0 | -29 | 190.1>163.0 | -21 | 0.01 | 93.3 | 102.2 | 88.4 | 88.4 | 9.56 | 12.5 | 4.75 | 4.76 |
| Trifloxystrobin | 99 | 25.56 | 409.1>186.1 | -19 | 409.1>145.1 | -44 | 0.01 | 97.5 | 98.5 | 91.7 | 95.9 | 8.66 | 4.04 | 3.27 | 1.32 |
| Triflumizole | 99 | 25.53 | 346.0>278.0 | -11 | 346.0>43.2 | -27 | 0.01 | 94.6 | 98.2 | 91.5 | 101 | 6.57 | 5.11 | 4.48 | 2.49 |
| Triflumuron | 99 | 24.22 | 359.0>156.1 | -17 | 359.0>139.1 | -30 | 0.01 | 91.9 | 84.2 | 89.3 | 97.6 | 15.7 | 14.8 | 9.7 | 2.11 |
| Triticonazole | 99 | 21.58 | 318.1>70.2 | -22 | 318.1>125.0 | -28 | 0.01 | 93.3 | 93.4 | 93.2 | 102.2 | 5.75 | 4.71 | 9.36 | 8.34 |
| Vamidothion | 99 | 10.81 | 288.1>146.1 | -12 | 288.1>118.0 | -22 | 0.01 | 90.4 | 97.3 | 85.1 | 93.2 | 5.54 | 8.1 | 6.74 | 5.77 |
| Zoxamide | 99 | 23.59 | 336.0>187.0 | -23 | 336.0>159.0 | -40 | 0.01 | 99.1 | 99.3 | 89.4 | 94.8 | 8.31 | 7.26 | 6.43 | 3.13 |
