# Supplementary material for: Validation and Simultaneous Monitoring of 311 Pesticide Residues in Loamy Sand Agricultural Soils by LC-MS/MS and GC-MS/MS, Combined with QuEChERS-Based Extraction
Source: Molecules. 2023 May 23;28(11):4268. doi: 10.3390/molecules28114268 (PMC10254206; doi:10.3390/molecules28114268)
Supplement: Supplementary file 1 [file molecules-28-04268-s001.zip › Supplementary Table S3.docx]

**Table S3.** Z-score results after the participation of the validated method in the Proficiency Test for the determination of pesticide residues in soil. (Laboratory code: 9973)

| **Analyte** | **z-scores** | |
| --- | --- | --- |
|  | **PT-PASS-2022-S1** | **PT-PASS-2022-S2** |
| **Azoxystrobin** |  | -1.49 |
| **Carbendazim** | -0.50 | -0.71 |
| **Clomazone** | <LOQ | -0.76 |
| **Cyproconazole** | <LOQ | <LOQ |
| **Difenoconazole** | <LOQ | <LOQ |
| **Diflufenican** | -0.58 | 0.00 |
| **Dimethachlor** | <LOQ | -0.79 |
| **Epoxiconazole** | -1.04 | -0.65 |
| **Fenpropimorph** | <LOQ | <LOQ |
| **Flufenacet** | <LOQ | <LOQ |
| **Fluoxastrobin** |  | <LOQ |
| **Flusilazole** | -0.43 | 0.62 |
| **Chlorotoluron** | <LOQ | <LOQ |
| **Chlorpyrifos** | <LOQ | <LOQ |
| **Imidacloprid** | <LOQ | <LOQ |
| **Linuron** | <LOQ |  |
| **Metconazole** | <LOQ | -0.08 |
| **Nanpropamide** | <LOQ |  |
| **Picoxystrobin** | <LOQ |  |
| **Prochloraz** | <LOQ | -1.10 |
| **Prometryn** | <LOQ |  |
| **Propiconazole** |  | <LOQ |
| **Spiroxamine** | 0.24 | 0.14 |
| **Tebuconazole** | -0.68 | -0.26 |
| **Terbuthylazine** | <LOQ | <LOQ |
| **Thiacloprid** | <LOQ | <LOQ |
| **Triadimenol** | <LOQ | <LOQ |
| **Trifloxystrobin** | <LOQ | <LOQ |
| **Atrazine** | <LOQ | <LOQ |
| **Metazachlor** | <LOQ | <LOQ |
