# Supplementary material for: Validation and Simultaneous Monitoring of 311 Pesticide Residues in Loamy Sand Agricultural Soils by LC-MS/MS and GC-MS/MS, Combined with QuEChERS-Based Extraction
Source: Molecules. 2023 May 23;28(11):4268. doi: 10.3390/molecules28114268 (PMC10254206; doi:10.3390/molecules28114268)
Supplement: Supplementary file 1 [file molecules-28-04268-s001.zip › Supplementary Table S4.docx]

**Table S4.** **Matrix effects assessed in LC-MS/MS and GC-MS/MS systems for all analytes. No matrix effect (ME) corresponds to |ΜΕ| <20%, Medium ME to 20%<|ΜΕ|<50% and Strong ME to |ΜΕ|>50%, respectively.**

| **Analytes** | **No ME^a^** | **Medium ME^b^** | **Strong ME^c^** |
| --- | --- | --- | --- |
| LC-MS/MS | | | |
| Methamidophos, formetanate HCl, aldicarb sulfoxide, butoxycarboxim, aldicarb sulfone, nitenpyram, Oxamyl, Carbendazim, Methomyl, Flonicamid, Thiamethoxam, thiabendazole, Monocrotophos, dicrotophos, clothianidin, fenuron, Dimethoate, dioxacarb, mevinphos, Vamidothion, 3-hydroxycarbofuran, acetamiprid, Mexacarbate, thiacloprid, trichlorfon, tricyclazole, Butocarboxim, ethirimol, aldicarb, oxadixyl, carbetamide, pirimicarb, thidiazuron, thiophanate methyl, bendiocarb, propoxur, imazalil, carbofuran, Metribuzin, tebuthiuron, carbaryl, carboxin, simetryn, monolinuron, fluometuron, chlorotoluron, Prometon, secbumeton, metobromuron, Methabenzthiazuron, propham, flutriafol, spiroxamine, forchlorfenuron, isoproturon, diuron, Metalaxyl, fenpropimorph, ametryn, cycluron, Methoprotryne, chlorantraniliprole, pyrimethanil, Azoxystrobin, furalaxyl, linuron, diethofencarb, Ethofumesate, methiocarb, fenamidone, ethiprole, Boscalid, paclobutrazol, promecarb, mandipropamid, Prometryn, flutolanil, mepronil, terbutryn, Methoxyfenozide, cyproconazole, myclobutanil, Triadimefon, mefenacet, bromuconazole, Chloroxuron, triadimenol, fluquinconazole, Fenarimol, spirotetramat, fenhexamid, iprovalicarb, Triticonazole, butafenacil, etaconazole, bupirimate, Epoxiconazole, tetraconazole, flufenacet, Fenbuconazole, diflubenzuron, Cyprodinil, rotenone, flusilazole, fenoxycarb, neburon, tebufenozide, dimoxystrobin, picoxystrobin, Diclobutrazol, spinosyn A, kresoxim methyl, penconazole, tebuconazole, benalaxyl, Zoxamide, propiconazole, prochloraz, hexaconazole, metconazole, clofentezine, spinosyn D,  Hydramethylnon, triflumuron, thiobencarb, spinetoram, benzoximate, pencycuron, difenoconazole, Triflumizole, ipconazole, indoxacarb, trifloxystrobin, clethodim isomer II, emamectin-benzoate b1a, furathiocarb, buprofezin, tebufenpyrad, temephos, piperonyl butoxide, quinoxyfen, pyriproxyfen, Metaflumizone, hexythiazox, etoxazole, propargite, flufenoxuron, fenpyroximate, spirodiclofen, chlorfluazuron, fenazaquin, pyridaben, amitraz, fipronil, fluazinam, imidacloprid, dimethomorph,  Pyraclostrobin, fludioxonil, hexaflumuron, lufenuron | 164 | - | - |
| Nuarimol, fluoxastrobin | - | 2 | - |
| GC-MS/MS |  |  |  |
| Di-allate-1, Diazinon, Fenoxycarb, Hexachlorobenzene, Pentachloroanisole, Propachlor, Trifluralin | 7 | - | - |
| 2,4'-Methoxychlor, 4,4'-Dichlorobenzophenone, 4,4'-methoxychlor olefin, Acrinathrin-1, alpha-Endosulfan, Ametryn, Azinphos-ethyl, Azinphos-methyl, Azoxystrobin, Benfluralin, beta-Endosulfan, Bifenthrin, Boscalid, Bromfenvinfos-methyl, Bromfenvinphos, Bromophos-ethyl, Bromopropylate, Bupirimate, Carbophenothion, Carboxin, Carfentrazone-ethyl, Chlorbenside, Chlorfenapyr, Chlorfenson, Chlorfenvinphos, Chlorobenzilate, hlorpyrifos, Chlorthal-dimethyl, Chlorthiophos-3, Chlozolinate, cis-Chlordane, cis-Nonachlor, cis-Permethrine, Cycloate, Cyfluthrin-1, Cypermethrin-1, Cyprodinil, delta-BHC, Deltamethrin-1 (Tralomethrin deg.-1), Dieldrin, Difenoconazole-1, Diflufenican, Dimethomorph-1, Diphenamid, Edifenphos, Endosulfan sulfate, Endrin, Endrin ketone, EPN, Epoxiconazole, Ethion, Etofenprox, Famoxadone, Fenamiphos, Fenarimol, Fenoxaprop-ethyl, Fenoxaprop-P-ethyl), Fenpropathrin, Fenson, Fenvalerate-1, Fipronil, Fluazifop-P-butyl, Fluchloralin, Flucythrinate-1, Fludioxonil, Flufenacet, Fluquinconazole, Fluridone, Flusilazole, Flutolanil, Flutriafol, gamma-BHC (Lindane), Hexazinone, Indoxacarb, Iodofenphos, Isodrin, Isopropalin, Kresoxim-methyl, lambda-Cyhalothrin, Leptophos, Mefenpyr-diethyl, Metalaxyl (Mefenoxam), Metazachlor, Methoxychlor, Metribuzin, MGK 264-1, Mirex, Myclobutanil, Napropamide, Nitralin, Nitrofen, Norflurazon, o,p'-DDD, o,p'-DDE, o,p'-DDT, Oxadiazon, Oxyfluorfen, p,p'-DDD, p,p'-DDE, p,p'-DDT, Paclobutrazol, Parathion-methyl, Pentachloroanisole, Pentachlorobenzene, perthane, Phenothrin-2, Phorate, Phosalone, Phosmet, Piperonyl butoxide, Pirimiphos ethyl, Pretilachlor, Procymidone, Prodiamine, Profenofos, Prometryn, Propargite-1, Propisochlor, Prothiofos, Pyrazophos, Pyridaben, Pyridaphenthion, Pyriproxyfen, Quinalphos, Quizalofop-ethyl, (Quizalofop-P-ethyl), Resmethrin-2 (Bioresmethrin), Sulfotep, Sulprofos, tau-Fluvalinate-1, Tebuconazole, Tebufenpyrad, Tefluthrin, Terbufos, Terbuthylazine, Tetrachlorvinphos, Tetradifon, Tetramethrin-2, trans-Chlordane, trans-Nonachlor, Triadimenol-1, Triazophos, Trifloxystrobin, Vinclozolin |  | 142 |  |
| Acetochlor, alpha-BHC, beta-BHC, Bromophos methyl, Clomazone, Coumaphos, Ethoprophos, Fenchlorphos, Fenthion, Fonofos, Isazofos, Lenacil, Malathion, Metolachlor (S-Metolachlor), Penconazole, Pentachlorothioanisole, Pirimiphos-methyl, Prochloraz, Propyzamide, Pyraclofos, Tolclofos-methyl, trans-Permethrine, Tri-allate, Triflumizole |  |  | 24 |
